# Intestinal Ischaemia and Perforation After Veno‐Venous Extracorporeal Membrane Oxygenation (VV‐ECMO) for Acute Exacerbation of Interstitial Lung Disease: An Autopsy Case

**DOI:** 10.1002/rcr2.70656

**Published:** 2026-06-19

**Authors:** Hiroka Miyagawa, Shigeo Hanada, Yasuro Miura, Hiroshi Nakahama, Yui Takahashi, Yuichiro Nei, Takahiro Mitsumura, Atsushi Miyamoto, Meiyo Tamaoka

**Affiliations:** ^1^ Department Of Respiratory Medicine, Respiratory Center Toranomon Hospital Tokyo Japan; ^2^ Okinaka Memorial Institute for Medical Research Tokyo Japan; ^3^ Department Of Pathology Toranomon Hospital Tokyo Japan

**Keywords:** acute exacerbation, interstitial lung disease, intestinal perforation, pulmonary embolism, veno‐venous extracorporeal membrane oxygenation

## Abstract

A 75‐year‐old man with interstitial lung disease was admitted with an acute exacerbation and severe hypoxemic respiratory failure. During intensive care management, he developed acute myocardial infarction, required veno‐venous extracorporeal membrane oxygenation and later underwent continuous haemodiafiltration for an acute kidney injury. A pulmonary embolism, gastrointestinal bleeding and intestinal perforation subsequently occurred, and the patient died of septic shock on day 40 of hospitalisation. An autopsy showed diffuse alveolar damage superimposed on a usual interstitial pneumonia pattern, intra‐alveolar haemorrhage, a subacute myocardial infarction, pulmonary arterial thrombi, a terminal ileal perforation with transmural necrosis and microthrombi in the intestinal wall. This case illustrates the difficulty of balancing thrombosis and bleeding during and after extracorporeal support for an acute exacerbation of interstitial lung disease and highlights the need for early suspicion of intestinal ischaemia in patients receiving extracorporeal membrane oxygenation who exhibit unexplained anaemia, gastrointestinal bleeding, worsening sepsis, or rising lactate levels, particularly during deep sedation.

## Graphical abstract

A patient with an acute exacerbation of interstitial lung disease developed a myocardial infarction, a pulmonary embolism and a fatal intestinal perforation after veno‐venous extracorporeal membrane oxygenation and continuous haemodiafiltration. This autopsy case highlights the difficulty of balancing thrombosis and bleeding and the need for early suspicion of intestinal ischaemia during deep sedation.

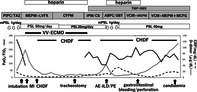

## 1 Introduction

Acute exacerbation of interstitial lung disease (AE‐ILD) may require invasive respiratory support, including extracorporeal membrane oxygenation (ECMO) in selected cases. However, critically ill patients receiving extracorporeal support are vulnerable to both thrombotic and haemorrhagic complications, and mesenteric ischaemia is a rare but catastrophic abdominal complication in this setting [1, 2]. We report an autopsy case of AE‐ILD complicated by a myocardial infarction, a pulmonary embolism and a fatal intestinal perforation after veno‐venous ECMO (VV‐ECMO) and continuous haemodiafiltration (CHDF).

## 2 Case Report

A 75‐year‐old man with angina pectoris, hypertension and alcoholic fatty liver disease had been evaluated 4 months earlier for progressive exertional dyspnoea and was diagnosed with interstitial lung disease. He presented with a worsening cough, mild haemoptysis, fever and dyspnoea. Chest computed tomography (CT) showed new bilateral ground‐glass opacities superimposed on pre‐existing fibrotic changes, and an AE‐ILD was initially suspected on the basis of this acute radiological deterioration and the overall clinical presentation (Figure 1A). Laboratory testing showed inflammation and a markedly elevated D‐dimer level. Because the bloody sputum also raised concerns regarding diffuse alveolar haemorrhage, high‐flow oxygen, piperacillin–tazobactam and methylprednisolone pulse therapy were initiated. Bronchoscopy with bronchoalveolar lavage was subsequently performed on hospital day 10, and a bloody bronchoalveolar lavage fluid supported the diagnosis of diffuse alveolar haemorrhage.

**FIGURE 1:**
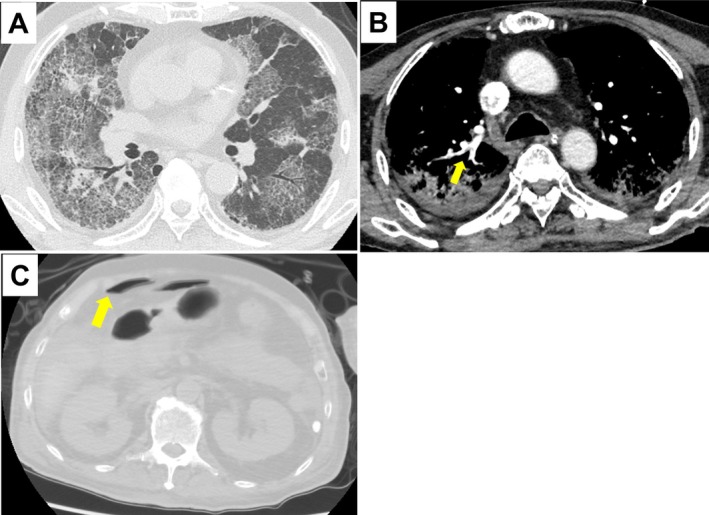
Radiological findings of an acute exacerbation, a pulmonary embolism and an intestinal perforation. (A) Chest computed tomography on admission revealed new bilateral ground‐glass opacities superimposed on fibrotic changes, consistent with an acute exacerbation of interstitial lung disease. (B) Contrast‐enhanced chest computed tomography on hospital day 22 showing an intraluminal filling defect in the right pulmonary artery, consistent with pulmonary embolism (arrow). (C) Abdominal computed tomography on hospital day 36 showing free intraperitoneal air, indicating gastrointestinal perforation (arrow).

Despite treatment, respiratory failure progressed, and invasive mechanical ventilation was required. On hospital day 4, ST‐segment elevation was observed on electrocardiography. Emergency coronary angiography revealed total occlusion of the seventh branch and severe stenosis of the second and third branches. Because of persistent findings indicating alveolar haemorrhage, dual antiplatelet therapy was withheld and percutaneous balloon angioplasty alone was performed. Before ECMO initiation, the patient showed profound hypoxaemia despite mechanical ventilation, whereas his blood pressure was 100–120/60–70 mmHg without vasopressor support, and the serum lactate level was 2.1 mmol/L. Although an acute myocardial infarction had occurred, refractory hypoxaemia rather than circulatory collapse was the dominant indication for extracorporeal support. Because the systemic circulation was considered sufficiently maintained with acceptable cardiac function on echocardiography, VV‐ECMO rather than veno‐arterial ECMO was selected. Systemic anticoagulation with continuous intravenous unfractionated heparin at 10,000–15,000 units/day was started on the same day and titrated according to serial activated clotting time (ACT) and activated partial thromboplastin time (aPTT) measurements. Although the target ranges are generally 50–70 s for aPTT and 160–180 s for ACT, they were reduced to 40–50 s and 140–160 s, respectively, depending on the severity of the bloody sputum attributed to alveolar haemorrhage.

CHDF for acute kidney injury was started on hospital day 5. Heparin was discontinued at the time of VV‐ECMO withdrawal. On hospital day 22, contrast‐enhanced chest CT demonstrated pulmonary embolism (Figure 1B), and continuous intravenous unfractionated heparin therapy was subsequently resumed. Because sepsis was suspected, broad‐spectrum antimicrobial therapy was escalated and *Candida albicans* and *Enterococcus faecium* were repeatedly isolated from respiratory or catheter samples; blood cultures later confirmed candidaemia. The clinical course was further complicated by progressive anaemia and gastrointestinal bleeding during the course of CHDF and after VV‐ECMO (Figure 2). During this period, the platelet count remained low, ranging from 60 × 10^3^/μL to 80 × 10^3^/μL, and coagulation‐related laboratory abnormalities included elevated D‐dimer levels that reached 10.9 μg/mL. The haemoglobin level gradually decreased to 6.4 g/dL and melena appeared, suggesting gastrointestinal bleeding. In total, the patient required transfusion of 24 units of red blood cells, 2 units of fresh frozen plasma and 10 units of platelets. On hospital day 36, CT revealed free intraperitoneal air without active contrast extravasation, indicating gastrointestinal perforation (Figure 1C). Because the patient had severe shock and multiorgan failure, surgical intervention was not feasible. He died of septic shock on day 40 of hospitalisation (Figure 2).

**FIGURE 2:**
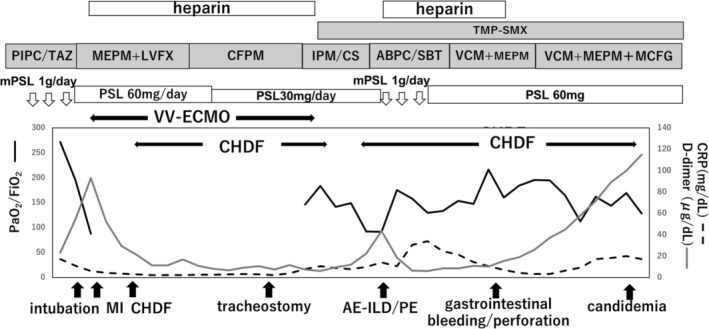
Clinical course. The patient developed an acute myocardial infarction on hospital day 4 during treatment for an acute exacerbation of interstitial lung disease, followed by VV‐ECMO for refractory hypoxaemia. Systemic anticoagulation with heparin was initiated on the day of VV‐ECMO initiation, discontinued at the time of VV‐ECMO withdrawal and resumed on hospital day 22 after a pulmonary embolism was diagnosed. Continuous CHDF for an acute kidney injury was started on hospital day 5. The subsequent course was complicated by gastrointestinal bleeding, an intestinal perforation and candidaemia. Changes in the PaO_2_/FiO_2_ ratio, CRP level and D‐dimer level are shown. Abbreviations: TMP‐SMX, trimethoprim‐sulfamethoxazole; PIPC/TAZ, piperacillin‐tazobactam; MEPM, meropenem; LVFX, levofloxacin; CFPM, cefepime; IPM/CS, imipenem‐cilastatin; ABPC/SBT, ampicillin‐sulbactam; VCM, vancomycin; MCFG, micafungin; mPSL, methylprednisolone; PSL, prednisolone; VV‐ECMO, veno‐venous extracorporeal membrane oxygenation; CHDF, continuous haemodiafiltration; MI, myocardial infarction; AE‐ILD, acute exacerbation of interstitial lung disease; CRP, C‐reactive protein; PaO_2_/FiO_2_, arterial oxygen partial pressure/fraction of inspired oxygen ratio.

An autopsy revealed diffuse alveolar damage superimposed on a usual interstitial pneumonia pattern, with intra‐alveolar haemorrhage and multiple pulmonary arterial thrombi. The left ventricle showed a subacute myocardial infarction. The terminal ileum showed a perforation with transmural necrosis, and histopathology demonstrated multiple microthrombi in the submucosal vessels adjacent to the perforated segment. Although a formal quantitative pathological assessment was not performed, the microthrombi were considered to be associated with marked intestinal wall injury. These findings suggested multifactorial thrombotic‐haemorrhagic complications with fatal intestinal ischaemia and perforation (Figure 3).

**FIGURE 3:**
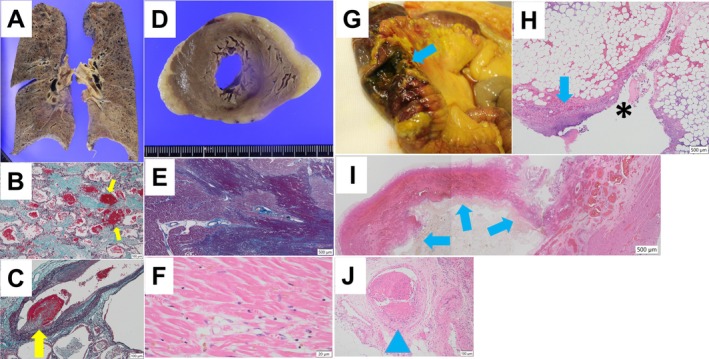
Autopsy findings demonstrating multiorgan thrombotic–haemorrhagic complications in the lung, heart and intestine. (A) Macroscopic appearance of the lungs showing bilateral honeycomb changes. (B) Masson's trichrome staining of the lung showing diffuse alveolar damage superimposed on fibrotic interstitial changes, with intra‐alveolar haemorrhage (arrows). (C) Masson's trichrome staining showing a thrombus within a pulmonary artery (arrow). (D) Macroscopic cross‐section of the heart showing necrotic changes in the interventricular septum. (E) Masson's trichrome staining of the myocardium showing fibrosis replacing necrotic myocardium. (F) High‐power view of the myocardium showing loss of cross‐striations with focal nuclear disappearance and pyknosis, consistent with subacute myocardial infarction. (G) Macroscopic appearance of the terminal ileum showing perforation (arrow) with surrounding discolouration. (H) Histopathological image of the perforated ileum showing transmural necrosis (arrow) and perforation (asterisk). (I) Histopathological image of the ileal wall showing marked congestion and necrosis (arrows). (J) Histopathological image showing a microthrombus in a submucosal vessel (arrowhead).

## 3 Discussion

Based on the present case, we could not conclude that AE‐ILD alone caused systemic coagulopathy. Rather, several interacting mechanisms likely contributed to the thrombotic–haemorrhagic complications. Severe systemic inflammation associated with AE‐ILD and critical illness may have promoted endothelial injury and a prothrombotic state, predisposing the patient to microthrombus formation. In addition, ECMO‐related circulatory alterations may have contributed to mesenteric hypoperfusion, and non‐occlusive mesenteric ischaemia may have been involved in multiorgan failure and circulatory vulnerability [3]. Anticoagulation was also inherently difficult, because continuous unfractionated heparin had to be initiated during VV‐ECMO, discontinued after ECMO withdrawal and subsequently resumed after the diagnosis of a pulmonary embolism, while the target ACT and aPTT ranges were reduced in response to bloody sputum attributed to alveolar haemorrhagic activity [4, 5]. Therefore, the autopsy findings of transmural intestinal necrosis with microthrombi were most consistent with a multifactorial mechanism involving both impaired mesenteric perfusion and thrombotic injury [3, 4, 5]. Although the thrombocytopaenia and coagulation abnormalities suggested a consumptive coagulopathic state, the available clinicopathological findings were not sufficient to establish overt disseminated intravascular coagulation. Rather, the intestinal microthrombi were interpreted as part of a multifactorial thrombotic process associated with systemic inflammation, critical illness, extracorporeal circulation and a disturbed haemostatic balance [3, 4, 5].

This case highlights two key clinical lessons. First, balancing thrombosis prevention and bleeding risk is particularly difficult during combined VV‐ECMO and CHDF [1]. Second, intestinal ischaemia and perforation may be overlooked during deep sedation because abdominal symptoms are often masked. In retrospect, intestinal ischaemia might have been suspected earlier when progressive anaemia, melena, worsening sepsis and, if available, rising lactate levels became evident before the detection of free intraperitoneal air. However, because the patient remained under deep sedation and was critically ill, abdominal symptoms and physical findings were difficult to assess. In this setting, earlier abdominal imaging may have been warranted once unexplained anaemia, gastrointestinal bleeding, worsening sepsis without a clear source, rising lactate levels, or abdominal distension became apparent. In patients receiving extracorporeal support, these findings necessitate prompt and careful reassessment and a low threshold for abdominal CT. Our case suggests that delayed recognition of gastrointestinal ischaemia in this setting may have fatal consequences [2, 6]. This case differs from previously reported cases of ECMO‐related intestinal ischaemia in several respects. First, it occurred during the management of an AE‐ILD requiring VV‐ECMO and CHDF rather than the more commonly reported contexts of cardiogenic shock or post‐cardiotomy support. Second, the clinical course was complicated by a concomitant myocardial infarction, a pulmonary embolism and gastrointestinal bleeding, highlighting a complex thrombotic–haemorrhagic milieu. Third, the autopsy confirmed transmural intestinal necrosis with microthrombi, providing clinicopathological evidence to support the diagnosis and its mechanistic interpretation. These features, together with the delayed recognition of abdominal complications during deep sedation, underscore the novelty and educational value of this case [2, 6].

## Author Contributions

All authors provided patient care. Hiroka Miyagawa, Shigeo Hanada, and Meiyo Tamaoka drafted and edited the manuscript, and selected the images. All authors contributed to the interpretation of data and critically reviewed and approved the final version of the manuscript. All authors met the ICMJE authorship criteria.

## Ethics Statement

Formal ethics committee review was waived for this single‐patient case report according to the institutional policy of Toranomon Hospital.

## Consent

The authors declare that written informed consent was obtained for the publication of this manuscript and accompanying images and attest that the form used to obtain consent from the patient complies with the Journal requirements as outlined in the author guidelines.

## Conflicts of Interest

The authors declare no conflicts of interest.

## Data Availability

Data sharing not applicable to this article as no datasets were generated or analysed during the current study.
